# Heart and vessels from stem cells: A short history of serendipity and good luck

**DOI:** 10.1002/bies.202400078

**Published:** 2024-06-05

**Authors:** Christine Mummery

**Affiliations:** ^1^ Dept Anatomy and Embryology Leiden University Medical Center Leiden The Netherlands

**Keywords:** cardiovascular disease, embryonic stem cells, heart development, induced pluripotent stem cells, vasculogenesis

## Abstract

Stem cell research is the product of cumulative, integrated effort between and within laboratories and disciplines. The many collaborative steps that lead to that special “Eureka moment”, when something that has been a puzzle perhaps for years suddenly become clear, is among the greatest pleasures of a scientific career. In this essay, the serendipitous pathway from first acquaintance with pluripotent stem cells to advanced cardiovascular models that emerged from studying development and disease will be described. Perhaps inspiration for later generations of stem cell researchers simply to follow whatever they find interesting.

## Graphical abstract

The history of pluripotent stem cell research spans more than 50 years: from teratomas, tumors in certain strains of mice and in young men, stem cells isolated from these tumors and embryos in in vitro culture, to human disease models for drug discovery, investigating toxicity and transplantation of differentiated human stem cells for tissue repair in regenerative medicine. Some of the key scientists in this story who crossed my own path are placed in context in this personal reflection. Image reproduced with permission from M. W. Lensch & C. L. Mummery (2013), Stem Cell Reports 1(1):5‐17.

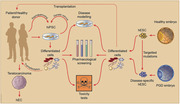

## THE BEGINNING

Trained as a biophysicist in the UK, my first encounter with the concept of differentiation came during my first postdoc at the Hubrecht Institute in the Netherlands. The subject was neuroblastoma cells, that form in the brain of mice and humans. These remarkable tumor cells grow extremely rapidly in culture but can also be induced to form massive networks of neurons simply by adding retinoic acid or DMSO.^[^ ^1^ ^]^ They then stop dividing and change from being malignant to benign within the matter of a week. We thought that by inducing differentiation, we might be able to cure this type of cancer in humans. This was based on the concept that carcinomas are caricatures of tissue renewal and noncytotoxic treatments could be based on growth factors that control physiological responses of normal cells such that they divide or differentiate. Whilst that did not work, we learnt a great deal about the relationship between the cell cycle, differentiation and the electrophysiological properties of neurons.^[^ ^2^ ^]^

Fast forward to a Cold Spring Harbor Laboratory meeting on teratocarcinoma stem cell biology in 1982. Teratocarcinomas are also caricatures of normal tissue, in this case of early embryos. First identified by Leroy Stevens in 1954 in certain strains of mice,^[^ ^3^ ^]^ they were shown by Barry Pierce to be expandable as ascites in the same mouse strain and, by cloning,^[^ ^4^ ^]^ to contain a multipotent stem cell population which he called embryonal carcinoma (EC). Almost every laboratory in the world studying teratocarcinoma or EC cells in mouse or humans was represented at that incredible Cold Spring Harbor meeting. Too junior to be selected, I was delighted when my group leader bought the conference proceedings “Teratocarcinoma Stem Cells,” edited by Lee Silver, Gail Martin and Sid Strickland^[^ ^5^ ^]^ a year later. This book was inspirational: it would for years be the “go to” reader for all you wanted to know about these stem cells. It contained contributions from Gail Martin and Martin Evans labs describing the first attempts to derive mouse embryonic stem cells from blastocyst stage embryos, the normal equivalent of mouse ECs, and human ECs derived from primary human teratocarcinomas. This was then one of the primary causes of death among young men. Loss of malignancy was here also discussed by Peter Andrews, Ivan Damjanov, and others as a reason to study differentiation.

Fascinated by these concepts and cells, I wrote to labs in Cambridge (Martin Evans with postdocs Liz Robertson, Alan Bradley, and Robin Lovell‐Badge) and Oxford (Chris Graham, John Heath, Austin Smith, Martin Pera) to ask if I might visit. Extremely welcoming answers so I immediately went to their labs. Culture of mouse ES cells^[^ ^6^ ^]^ in Cambridge directly on the bench, no sterile hoods needed apparently if you were careful. Then in Oxford, cultures of PC13EC cells which could form endoderm and human Tera2 EC cells^[^ ^7^ ^]^ which could form neurons if given retinoic acid. But many EC lines are not actually “pluripotent” and are often referred to as nullipotent, since they needed retinoic acid to differentiate at all. Mouse ES cells, however, were pluripotent, evidenced by their ability to integrate into early embryos and differentiate into derivatives of all three germ layers, in contrast to ECs. They were, however, little used for studying directed differentiation in vitro and were difficult to obtain. John Heath suggested that what I might be looking for was P19EC cells which could form both neurons and beating cardiomyocytes.^[^ ^8^ ^]^ These cells were derived by Mike McBurney from teratomas that formed after postimplantation mouse embryos were transferred to an extrauterine site, a process he described in the CSHL Teratocarcinoma Stem Cells book. I returned to the Hubrecht Institute, with promises not only of P19 and PC13 EC cells but also mouse ES cells. By that time, we had also collected F9EC cells. A new research phase was opened.

## FIRST RESEARCH ON PLURIPOTENT STEM CELLS

Starting out was truly just “having a go” with papers available mostly from labs experienced in working with mouse embryos. F9, P19 and PC13 EC cell lines all grew without “feeder cells” to keep them in an undifferentiated state but mouse ES cells had to be grown in co‐culture with growth inactivated fetal fibroblasts. F9 cells^[^ ^9^ ^]^ differentiated well to different types of endoderm using a protocol from Michael Wiles; PC13 also formed endoderm and were particularly easy to synchronize in the cell cycle simply by shaking off the loosely attached mitotic cells. And for P19 EC cells, using either DMSO or retinoic acid, would make them form neurons or beating cardiomyocytes. How amazing was that for a physicist! All based on landmark papers from these labs. Mouse ES cells were a bit more challenging though as we needed to produce the feeder fibroblasts from fetal mice quite regularly. The differentiated derivatives from P19EC cells turned out to grow quite well so we cloned them and made a whole series of differentiated cells lines that could be expanded for long periods in culture and all looked morphologically different: epithelial, mesenchymal or endoderm‐like, with corresponding markers.^[^ ^10^ ^]^ We tested many of these for their ability to support undifferentiated growth of mouse ESCs and a few did. But remarkably, on several that had visceral endoderm features, the mouse ESCs started to form robustly beating heart cells. Serendipity resulted in a small “Eureka moment.” In a developmental biology lab with many studies on *Xenopus Laevis* and *Axolotl*, it did not take us long to realize we were likely recapitulating signals from endoderm that are essential for formation of the heart from mesoderm in development. The embryo was proving to be our guide in directing differentiation. Over the subsequent years we delved deeply into the wnt, bone morphogenetic protein and activin signaling pathways, noting when and where they are expressed in development and modulating them in mouse ES cells using siRNA knockdown. To really understand underlying mechanisms though, we needed to be able to make chimeric mice and for that, I went with my tech to Colin Stewart's lab at the EMBL in Heidelberg. Then, isolated on a hill among vineyards, he taught us to make “aggregation chimeras” (by co‐culturing mouse ESCs with 8‐cell stage mouse embryos), sterilize male mice, make pseudopregnant females and transfer the chimeric embryos to the uterus. He said it needed a steady hand so we had to make all of the chimeric embryos before our first morning coffee!

Back to the lab and euphoria when a month or two later, our first brown and white chimeric pups were born. The animal house keeper just said “there are some very strange mice in your cages: piebald!” The reason to do this experiment in the first place though was to see what happened if we knocked down different transforming growth factor (TGF) ß receptors in mouse ESCs and let them form chimeras. Essentially, removing TGFß‐receptor type II, which binds TGFß ligand, resulted in almost complete loss of vasculature in the yolk sac, and was embryonic lethal by day 9.^[^ ^11^ ^]^ This further anchored our interest in the cardiovascular system, how it forms and why it loses function in development and later life.

## HUMAN PLURIPOTENT STEM CELLS

While the mouse work was ongoing in our lab, we were approached by a urologist and surgeon working on teratocarcinoma in humans. The surgeon was from the adjacent military hospital and the most common malignancy he saw among young soldiers was teratocarcinoma. It was often fatal unless pathology showed it contained no stem cells; it was then classified as a benign teratoma. Upon removal from the testes, these extraordinary tumors often contained complex structures that resembled an embryo: hair, skin, muscle, fat, even teeth, clearly recognizable but without spatial organization. The surgeon was interested to know whether stem cell differentiation in situ would turn the teratocarcinomas into teratomas. Back to Oxford where Martin Pera was the expert at deriving stem cell cultures from the primary testis tumors. With his help, we derived several new human teratocarcinoma stem cells and with grants from the Netherlands Cancer Foundation, attempted to induce differentiation as a potential cure. However, soon after, cis‐platinum was identified as a potent killer of stem cells and rapidly became the therapy we and patients hoped for. Our grant ended though, since the cancer was curable and nearly all young men with this malignancy these days survive. Back to our work in mice, trying to direct differentiation and understand the underlying mechanisms controlling this in development.

**FIGURE 1:**
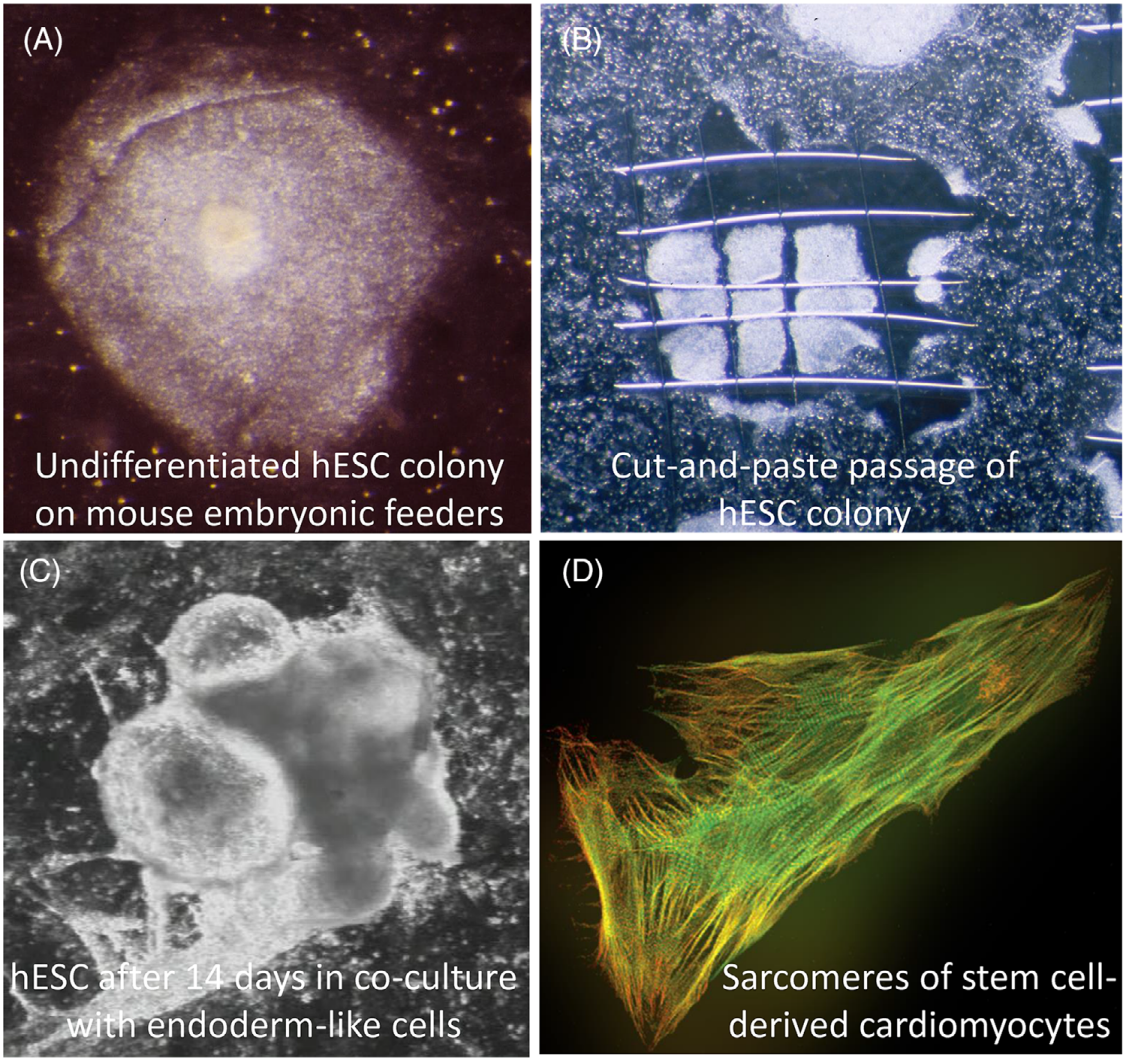
From undifferentiated cells to cardiomyocytes (anno 2001).^[^ ^15^, ^17^, ^34^ ^]^ (A) human embryonic stem cell (hESC) maintained as colonies on irradiated mouse embryonic fibroblasts (MEFs) as “feeder cells, (B) Colony of hESC “cut” into small pieces using glass needles. Each piece transferred to new MEFs or to endoderm‐like cells for differentiation, (C) 14 days after transfer to endoderm co‐cultures, hESC formed beating heart cells. (D) Staining of sarcomeres in pluripotent stem cell‐derived cardiomyocytes. (courtesy Richard Davis, LUMC), Adapted from refs. [15, 17, 34].

A few years later though, our second chance came to work on human stem cells. James Thomson derived nonhuman primate ES cells then a short time later, the first human embryonic stem cells (hESC) from the inner cell mass of blastocysts surplus to reproductive use in fertilization (IVF).^[^ ^12^ ^]^ Arif Bongso, gynecologist in Singapore, had perhaps established the first cultures from the inner cell mass of blastocysts slightly earlier but without experience in stem cell culture, he was challenged to create permanent cells lines. Working with Alan Trounson, Ben Reubinoff and Martin Pera, all then in Australia, however, derivation of hESC lines was successful and the second report was published.^[^ ^13^ ^]^ This was incredibly exciting for my lab, not only because the earlier interactions with the group prompted a generous offer to share the lines with us, but also because the Dutch parliament was about to vote on a new Embryo Law that would allow derivation of hESC in the Netherlands. It was controversial as in many countries, but Els Borst, the minister under whom the law fell was pro, and suggested we take up the offer. There was no law against importing any cells lines, including hESC, and she felt this discrepancy (import of hESC and research allowed, but no *de novo* derivation) would tip the balance in favour. It did and since 2002 it has been permissible in the Netherlands, with the right consents, to derive hESC from surplus IVF embryos.^[^ ^14^ ^]^ My lab derived the first five lines in 2003.^[^ ^15^ ^]^ This success was very much attributable to the meticulous training we received in Australia from Martin Pera: the right culture medium, feeder cells, “cut‐and‐past” passage of small cell clumps, skills no longer necessary but essential at that time to prevent “spontaneous” differentiation and loss of the pluripotent stem cell colonies (Figure 1A, B).

The main, in fact only, experiment I wanted to do was to grow hESC colonies on the visceral endoderm cells from P19EC we had used years before for mouse ESC cultures. Would the hESC also differentiate to cardiomyocytes? To date, they had only been reported to differentiate in vitro to neurons. Two weeks later, another “Eureka moment”: robustly beating areas of cardiomyocytes in the hESC colonies in co‐culture with the visceral endoderm‐like cells. By chance, Valentine's Day February 14th 2001 (Figure 1C, D). Our history of electrophysiology on neurons from neuroblastoma meant we were soon able to confirm that these cells indeed, electrophysiologically resembled ventricular‐like cardiomyocytes, albeit immature, and that they were connected to each other by gap junctions as in real myocardium of the heart. Kehat et al.^[^ ^16^ ^]^ published this first describing a method based on allowing the hESC to form “cystic embryoid bodies” which likely contained endodermal cells but soon after, we described our co‐culture method^[^ ^17^, ^18^ ^]^ showing signals indeed came for visceral endoderm. In the decades that followed we (and others) refined this to become feeder cell‐independent and serum‐free, the undifferentiated hESCs amenable enzymatic passage and timed addition of defined growth factors and/or small molecules sufficient in the absence of visceral endoderm to induce mesoderm then cardiac specification, eventually forming highly enriched cultures of cardiomyocytes.^[^ ^19^ ^]^ Whilst our earliest protocols yielded differentiated cultures with just 5% cardiomyocytes, our current protocols generally yield >90%.

## RECOGNIZING DIFFERENTIATED CELLS

It needed little imagination to guess that the rhythmically beating cells in culture with striated sarcomeres, evident even under phase contrast microscopy, were cardiomyocytes. Electrophysiology had confirmed it. The same was true for neurons: long neurite‐like structures growing out of large cell bodies and ending in what looked like growth cones. Electrophysiology confirmed. But what about the many other differentiated cells that formed from hESC: how did we know what they were? The developing embryo was our guide. In the beginning it was largely knowing which proteins were expressed in which cells in a developing mouse, looking to see if there were a human antibody (or make them as Peter Andrews did) and staining our differentiated cell cultures. In this way, many researchers identified different types of neurons, visceral endoderm, early liver‐like cells, and a multiplicity of others. The majority, however, remained unidentified. It would take many years and the collection and analysis of large amounts of data before we could do what we do today, that is single cell RNA sequencing. scRNAseq of cells from differentiating hPSCs and comparing this with data from (human and mouse) embryos at different stages of development now allows us to state with fair accuracy what we have in our cultures. Many more cell types than we ever dreamed of, although should have expected since they are pluripotent. Complemented with vast datasets from other ‐omics technologies, it is clear that cells can be generated from most organs and, with knowledge on how to recognize them, it has been possible to develop protocols that drive their directed differentiation, most efficiently if, in addition to using “markers” we use “reporter cell lines” to identify differentiated cells live in culture. One of the first we co‐developed with colleagues Dave Elliott, Andrew Elefanty and Ed Stanley was a hESC reporter line for cardiomyocyte differentiation using the cardiac transcription factor NKX2.5 to drive GFP expression.^[^ ^20^ ^]^

## ENTER: HUMAN INDUCED PLURIPOTENT STEM CELLS (hiPSC)

**FIGURE 2:**
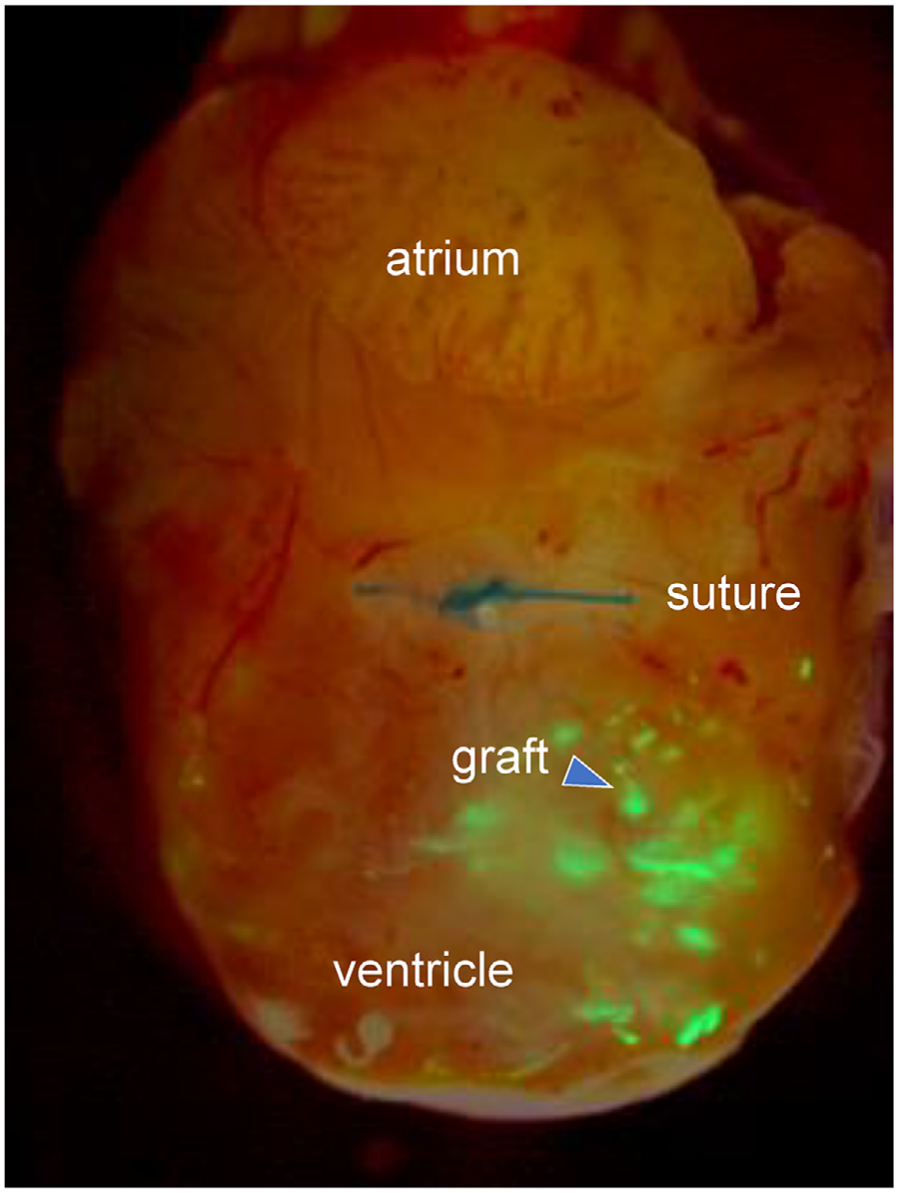
Transplantation of human embryonic stem cell (hESC)‐derived cardiomyocytes to mouse heart after myocardial infarction (anno 2008).^[^ ^21^ ^]^ Myocardial infarction in mouse heart induced by closure of descending aorta in the heart (suture). hESC‐cardiomyocytes carrying a genetically encoded GFP reporter injected into the ventricle of the heart and examined 14 days later. The GFP expressing cells are evident as a graft in the infarct zone in the ventricle. From van Laake et al. (2008).^[^ ^21^ ^]^

Despite the excitement surrounding hESC‐derived cardiomyocytes (hESC‐CMs) for cardiac repair, many countries had ethical concerns on the embryonic origin of hESC. This was a significant challenge for carrying out research, particularly in Europe and in NIH funded labs. Whilst my lab carried out extensive and successful studies transplanting hESC‐CMs in mice that had undergone experimental myocardial infarction (Figure 2),^[^ ^21^ ^]^ and the research was permitted in the Netherlands, I nevertheless had concerns about the enormous resources that would be required to bring hESC‐CMs to the clinic for heart repair. This was in 2007, the heyday of the “adult stem cell hype”: everything from bone marrow cells, “mesenchymal stem cells,” “cardiac stem cells” were being injected not only in animals but also in patients with myocardial infarction or heart failure. This eventually did not work. This area of research collapsed a decade later with the retraction of many tens of articles that were considered not only inconsistent but also fraudulent.^[^ ^22^ ^]^ Researchers like Chuck Murry, Loren Field and Irv Weismann, had years earlier pointed out the artefacts reported in many of these papers but the self‐cleansing mechanism in this particular field was slow to work, in part driven by the strong wish to have an ethically acceptable alternative to hESC.

Deciding essentially to give up our transplantation studies, I thought we might better focus on creating human cardiac models for detecting cardiotoxicity or understanding genetic diseases of the heart by introducing genetic mutations that caused cardiac arrhythmias. In both cases, arrhythmias caused by abnormal electrical action potentials could be fatal. This was a serious medical concern. Regarding the adverse effects of drugs, several were withdrawn from the market when patients underwent sudden cardiac death. Clearly, animal studies were not picking up these risks.

Encouraged by Kit Parker, Ken Chien, and Doug Melton in Boston, I applied for, and was awarded, a Radcliffe Fellowship for a sabbatical to learn more about engineering cardiomyocytes to make them more mature and create better human heart models. Incredibly inspiring to be part of the Harvard Stem Cell Institute and Mass General Hospital and wonderful to be at the bench again, bioprinting cardiomyocytes. Good luck once more though: whilst I was there, Shinya Yamanaka published his paper on hiPSC,^[^ ^23^, ^35^ ^]^ created by reprogramming normal somatic cells in the body to a pluripotent state without using embryos. This seemed to go through the Harvard Stem Cell Institute almost as a shock wave as the institute had been established in part to do hESC research independent of NIH funding. What now? Continue with hESC or set up hiPSC? The latter appeared the decision and in no time George Daley's lab had derived the first hiPSC lines from patients. Incredibly generous, he shared his lab's reagents and bench protocols and upon returning home we were able to become the first lab in the Netherlands generating hiPSC.^[^ ^24^ ^]^ Moreover, all of the protocols we had developed for hESC also worked for hiPSC. A new door opened: we not only could generate heart disease cell lines by homologous recombination in hESC^[^ ^25^ ^]^ but could also now derive them directly from patients.^[^ ^25^ ^]^ Since these first retroviral delivery systems were used for the reprogramming, there have been many technical advances but most recent are what are referred to as nonintegrating methods, in which the reprogramming factors are delivered as episomal vectors, plasmids, mRNAs and others but, importantly, do not integrate into the host genome. We have used all of these methods but independent of how reprogramming was done, building heart disease models is largely what we have done since: maturing the cardiac derivatives of hPSCs, developing analytical methods to quantify phenotypes robustly, finding ways to understand underlying disease mechanisms and screening drugs on hiPSC derivatives for their adverse and beneficial effects.^[^ ^26^, ^27^ ^]^ Perceived contributions of human stem cell‐based model systems in general to human health, particularly in the disease modeling and toxicity space, is illustrated by the recent FDA Modernization Act passed by the US Senate (December 2022). This states the FDA will “establish a process for the qualification of nonclinical testing methods to reduce and replace the use of animals in nonclinical research, improve the predictivity of nonclinical testing methods, and reduce development time for a biological product or other drug.” As this begins to be implemented, significant reductions in the costs and time of drug development may be expected, and the effectiveness and precision of the resultant drugs may greatly improve.

## NON‐CONTRACTILE CELLS IN THE HEART

Aside from contractile cardiomyocytes, the heart also contains several other cell types, all crucial for normal heart function. Every cardiomyocyte touches a blood vessel, for example, to ensure adequate nutrition, oxygen and removal of waste. In fact, around 70% of the adult heart is composed of stromal cells: cardiac endothelial cells making up the blood vessels and cardiac fibroblasts, providing the structural scaffold. Both cell types express markers typical of all other endothelial cells or fibroblasts in the body, yet they also have organ specific features, expressing transcription factors and other genes normally associated with cardiomyocytes. In our attempts to create mature hPSC‐cardiomyocytes we co‐cultured these three cardiac cell types in small, self‐aggregating structures we termed cardiac microtissues.^[^ ^28^ ^]^ We discovered that within these microtissues, cardiac fibroblasts formed gap junctions with cardiomyocytes and endothelial cells were in dialogue with both cell types, ultimately raising intracellular cyclic AMP levels within the cardiomyocytes. Excitingly, the cardiomyocytes rapidly showed features of postnatal cardiomyocytes: higher electrical action potentials, better organization of the contractile sarcomeres and even t‐tubules, that increase significantly in number after birth to control calcium handling. We were able to show by systematically replacing each cell type in the microtissues individually with a mutant variant that it was not always the cardiomyocytes that were responsible for causing arrhythmias but, in the case of arrhythmic cardiomyopathy for example, mutant cardiac fibroblasts could essentially be the disease “culprit”.^[^ ^27^ ^]^ We have most recently been able to fully automate this process using robotics and currently carry out screens to discover new antiarrhythmic drugs.

The endothelial cells proved fascinating as well. In this case though, we found that just differentiating the hiPSC from patients in regular 2D culture was not always sufficient to see disease phenotypes. One example is a condition called hereditary hemorrhagic telangiectasia (or HHT). This is caused by mutations in genes associated with the TGF beta signaling pathway. HHT patients suffer from chronic and profound nosebleeds and studies in mice suggested that the mural cells did not associate sufficiently with the endothelial cells so that vessels were unstable. We differentiated endothelial and mural cells from HHT hiPSC and after 2 years work, we could not find much wrong with their interaction in almost every assay we tried. Then, Organs‐on‐Chip entered our lab, through chance discussions with tissue engineer Albert van den Berg. In these models, hiPSC endothelial and mural cells “self‐organized” under microfluidic flow: they formed hollow tubular networks with lumens large enough for cells to flow through. To our delight and surprise, we could then see the phenotype we had hoped for: poor interaction between outer mural‐ and inner endothelial cells and “leaky” vessels.^[^ ^29^ ^]^ Again here, a great opportunity to create human disease models in which to screen for drugs that could be “repurposed” – intended to treat one disease but effective in another – or identify compounds that could be further developed as drugs.

## WHERE NOW?

It might seem from this narrative that we have a “done deal” in understanding cardiac and vascular disease, finding drugs to treat it in every patient without side‐effects and finding out why some only patients develop symptoms of a genetic disease using either hESC or hiPSC. Unfortunately, we do not. Among the multiple challenges that remain include: directing maturation of differentiated derivatives beyond the fetal‐ or postnatal state to middle‐ and old age, when most diseases develop; ensuring assays are reproducible within and between labs; protocols are effective across different hPSC lines and that all of this can be done at medium to high throughput to screen compound libraries for potential therapeutics. We can indeed now produce large numbers of differentiated cells (in bioreactors for example) from hPSC lines and this allows many different compounds in principle to be screened. But the problem here is that end‐users, from academia and biotech to pharma would like to be able to test hiPSC from many individuals and genetic/ethnic backgrounds with just few compounds in a more precision medicine approach. These are areas in which we and others are currently investing.

Several original methods have been reported that drive maturation of hPSC‐derivatives. For cardiac cells, this has ranged from generating engineered heart tissues (EHTs) and Biowires that force cyclic stretch and strain or allow contraction against resistance, changing the energy substrates from glucose‐based as prior to birth to fatty acid‐based, as postnatally during lactation, adding hormones and growth factors to culture medium or growing multiple cardiac stromal cells in 3D with cardiomyocytes (reviewed in ref. [30]. However, there are still no models that resemble the aging human heart.

Developing reproducible and scalable platforms with companion analytics is another important focus area, several groups, including our own, now working on robotics systems for this purpose. Generating hiPSC lines from many individuals, differentiating them to the required cell types and using imaging or AI to screen phenotypes rapidly and round‐the‐clock seems to be where much of current work is going. Not only are organoid models from hPSCs becoming as versatile as those from adult stem cells, they also allow immune cells – a major trigger of diseased states‐ and stromal cells like the vasculature to be obtained isogenically and incorporated into the tissue model of choice. Moreover, microfluidics is an important feature of multiple tissue types, recapitulating blood flow or lymphatic fluid drainage. Organ‐on‐Chip systems incorporate microfluidic channels that are often lined with primary‐ or hPSC‐derived vascular endothelial cells. The outcomes are increasingly realistic representations of what happens in normal and diseased human tissues.^[^ ^31^, ^32^ ^]^ Whilst it is most unlikely that animal experiments will ever be replaced, we are poised for an era in which realistic human models of health and disease will be used for drug discovery, far beyond their increasing applications in regenerative medicine. If regulators accept at least some of these models because we have demonstrated they are robust, reproducible and predictive,^[^ ^33^ ^]^ precision medicine and accurate prognosis may for many patients be just around the corner.

## CONFLICT OF INTEREST STATEMENT

The author declares no conflicts of interest.

## Data Availability

Data sharing is not applicable to this article as no new data were created or analyzed in this study.
